# A pilot study investigating the effect of serum 25-hydroxyvitamin D on device-estimated sleep metrics in young black women: a single-arm open-label clinical trial

**DOI:** 10.1093/sleepadvances/zpag096

**Published:** 2026-09-01

**Authors:** Michele N D’Agata, Krista M Szymanski, Freda Patterson, Mackenzie L Rattigan, Alexs A Matias, David G Edwards, Christopher R Martens, Sushant M Ranadive, Melissa A Witman

**Affiliations:** Department of Kinesiology and Applied Physiology, College of Health Sciences, University of Delaware, Newark, DE, United States; Department of Kinesiology and Applied Physiology, College of Health Sciences, University of Delaware, Newark, DE, United States; Department of Health Behavior and Nutrition Science, College of Health Sciences, University of Delaware, Newark, DE, United States; Department of Kinesiology and Applied Physiology, College of Health Sciences, University of Delaware, Newark, DE, United States; Department of Kinesiology and Applied Physiology, College of Health Sciences, University of Delaware, Newark, DE, United States; Department of Kinesiology and Applied Physiology, College of Health Sciences, University of Delaware, Newark, DE, United States; Department of Kinesiology and Applied Physiology, College of Health Sciences, University of Delaware, Newark, DE, United States; Department of Kinesiology, School of Public Health, University of Maryland, College Park, MD, United States; Department of Kinesiology and Applied Physiology, College of Health Sciences, University of Delaware, Newark, DE, United States

**Keywords:** vitamin D, sleep, Actigraphy, black women

## Abstract

**Study Objectives:**

Vitamin D deficiency has been linked to poor sleep health outcomes in the general population. Black women (BLW) experience a higher prevalence of vitamin D deficiency and short and disturbed sleep compared with other races. Therefore, this study tested cross-sectional associations between serum 25-hydroxyvitamin D concentration ([25(OH)D]) and sleep health estimates in young adult BLW, followed by an 8-week vitamin D supplementation intervention.

**Materials and Methods:**

Forty-three 18-30-year-old BLW underwent blood sampling for serum [25(OH)D]. Sleep metrics were derived from 14-days of wrist accelerometry. Thereafter, participants with [25(OH)D] <30 ng/ml (N = 16) completed an 8-week vitamin D3 supplementation intervention (5000 IU/day) and repeated blood sampling and sleep monitoring.

**Results:**

Linear regressions demonstrate significant cross-sectional associations for [25(OH)D] with sleep efficiency (β = 0.11, *p* = .03; %) and sleep onset standard deviation (SOSD) (β = -0.53, *p* = .04; minutes), but no associations with sleep duration or sleep duration standard deviation (*p* > .18). Among intervention participants, paired-sample *t-*tests demonstrate a significant increase in [25(OH)D] (16.8 ± 7.0 vs. 55.3 ± 10.0, ng/ml; *p* < .001), however, no changes were observed among actigraphy-derived sleep variables (all *p* > .21).

**Conclusions:**

[25(OH)D] was cross-sectionally associated with sleep efficiency and SOSD, however, intervention results from this pilot study do not demonstrate a beneficial effect of vitamin D on several device-estimated sleep metrics in young BLW.

**Clinical Trial Registration:**

Effects of Vitamin D on Cardiovascular Health in Black Women, https://clinicaltrials.gov/study/NCT05656742?locStr=Delaware&country=United%20States&state=Delaware&cond=Vitamin%20D%20Deficiency&term=blood%20pressure&intr=Vitamin%20D&rank=1 Registered on ClinicalTrials.gov ID NCT05656742.

## Graphical Abstract

**Graphical Abstract:**
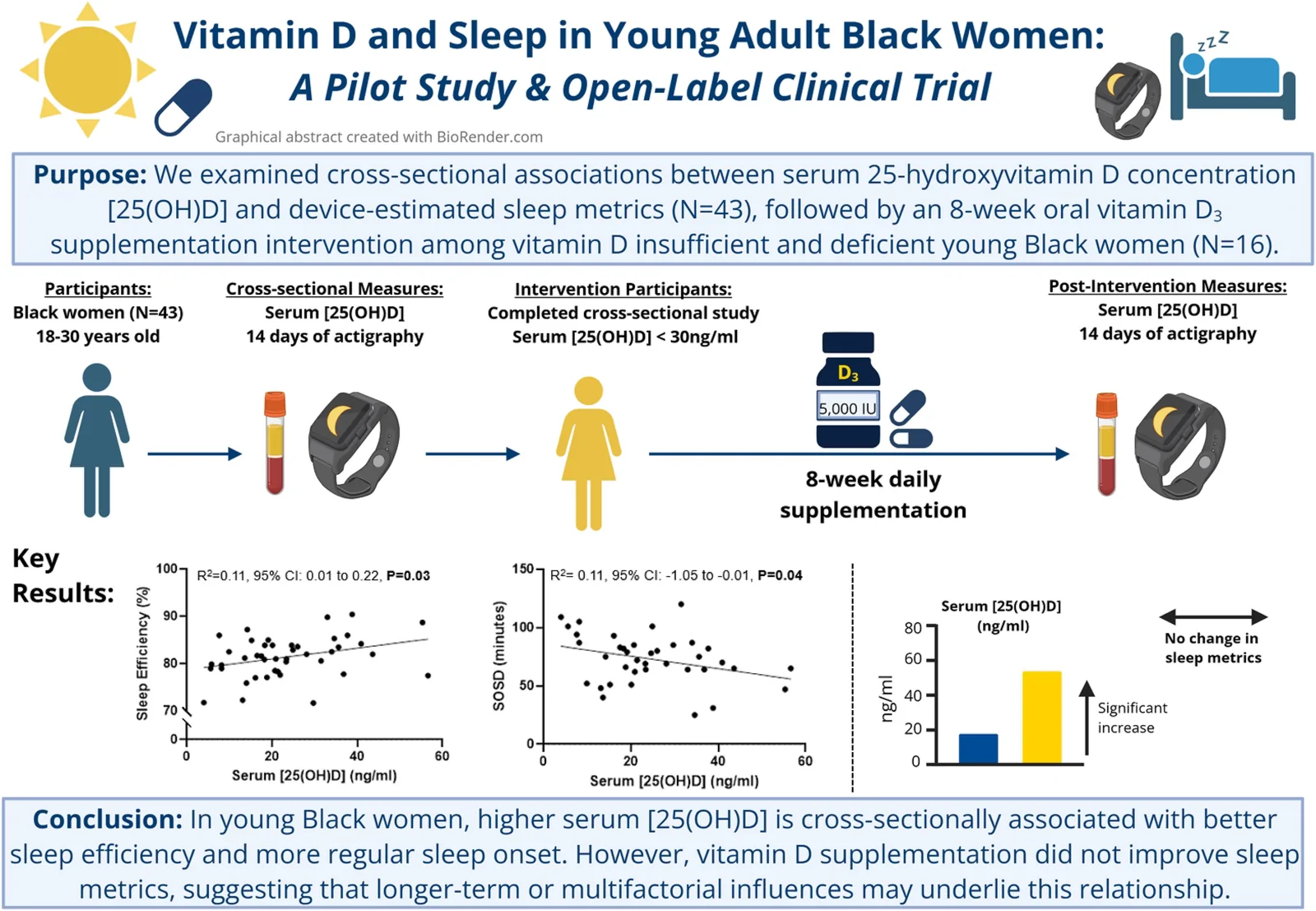

**Statement of Significance**

Many U.S. Black women (BLW) have suboptimal serum vitamin D concentrations ([25(OH)D]), which has been associated with poor sleep health in the general population. We examined the cross-sectional association between [25(OH)D] and device-estimated sleep health metrics followed by an oral vitamin D supplementation intervention in young BLW. We observed significant cross-sectional associations between [25(OH)D] and both sleep efficiency and sleep onset regularity. An 8-week oral vitamin D supplementation intervention significantly improved [25(OH)D] concentrations among vitamin D insufficient/deficient participants. However, acutely increasing [25(OH)D] did not improve sleep metrics, suggesting that the relationship between vitamin D status and sleep health may reflect longer-term physiological processes or broader behavioral and environmental influences rather than short-term causal effects of circulating vitamin D concentrations.

## Introduction

Poor sleep health is a major clinical and public health concern associated with a variety of adverse health outcomes including cardiometabolic diseases [1] and increased prevalence of cardiovascular and all-cause mortality [2]. Healthy adults are recommended to achieve ≥7 hours of sleep per night, with ≥85% sleep efficiency (percentage of time spent asleep while in bed) during each sleep period to minimize cardiovascular disease (CVD) risk [3, 4]. In addition, irregular nightly sleep duration and sleep-onset timing are independently associated with increased risk of cardiovascular events [5]. Despite this, one in three U.S. adults report not meeting sleep duration recommendations [6], and of added concern is that Black adults are more likely to experience short sleep duration, poorer sleep quality, and more irregular sleep duration and timing compared with other races, independent of age [6–9]. Moreover, the negative effects of poor sleep on cardiovascular risk may be greater in women compared with men [10, 11].

Black women (BLW) ≥ 20 years have the highest total CVD prevalence compared with both men and women of other racial and ethnic groups [6]. Evidence also indicates that young adult BLW are most likely to report short sleep duration (< 7 hours per night) and least likely to report adequate sleep duration (7-8 hours per night) compared with both non-Hispanic White women (WHW) and Hispanic women [7]. Using 14-days of wrist-actigraphy, our laboratory reported lower sleep efficiency and more irregular sleep duration and sleep timing among young adult BLW compared with young adult WHW [12]. Potential contributors to the observed racial disparities in sleep health among young women are largely understudied, but have been partly attributed to various social, cultural, and environmental factors based on evidence from large population-based studies [13]. Still, limited studies examine potential modifiable contributors to poor sleep health in young BLW.

Vitamin D insufficiency and deficiency (serum 25-hydroxyvitamin D concentrations ([25(OH)D]) <30 ng/ml) disproportionately affect an estimated 92% of BLW, likely due to low dietary vitamin D intake and a high concentration of eumelanin pigment in the skin [14, 15]. Mechanistic evidence suggests that vitamin D may influence sleep by aiding in the activation of the melatonin pathway [16], or by aiding in the synchronization of circadian clock gene expression [17]. Observational studies have demonstrated positive associations between [25(OH)D] and device-estimated sleep metrics including sleep duration in Black older adults [18] and sleep efficiency in White older men [19], and our laboratory and others have recently extended similar findings to generally healthy young to middle-aged adults [20, 21]. Importantly, experimental studies further demonstrate that increasing circulating [25(OH)D] via oral vitamin D supplementation improves self-report measures of daytime sleepiness and fatigue in healthy adults [22, 23]. However, no studies to date have investigated the effect of vitamin D supplementation on device-based (objective) estimates of habitual sleep health including sleep duration, sleep efficiency, or sleep regularity. The effect of vitamin D on sleep in young BLW has not been investigated and is especially relevant as they represent a population at high-risk for both vitamin D deficiency and sleep disturbances.

Therefore, the first aim of this pilot study was to cross-sectionally evaluate associations between serum [25(OH)D] and device-estimated sleep metrics including sleep duration, sleep efficiency, sleep duration standard deviation (SDSD), and sleep onset standard deviation (SOSD) in young adult BLW. The second aim was to subsequently evaluate the effect of an 8-week oral vitamin D supplementation intervention on device-estimated sleep metrics among BLW with [25(OH)D] < 30 ng/ml. Cross-sectionally, we hypothesized [25(OH)D] would be positively associated with sleep duration and sleep efficiency and inversely associated with SDSD and SOSD. We further hypothesized 8 weeks of oral vitamin D supplementation would increase [25(OH)D], sleep duration, and sleep efficiency, and decrease SDSD and SOSD as compared to baseline among BLW with [25(OH)D] <30 ng/ml.

## Materials and methods

This study was approved by the Institutional Review Board at the University of Delaware (IRB Study No. 1957713) and was registered with ClinicalTrials.gov (Study Identifier: NCT05656742). This study was conducted in accordance with the ethical standards of the *Declaration of Helsinki*, and written informed consent was obtained from all participants.

### Participants

Participants were recruited from the University of Delaware and the surrounding Newark, DE region and included apparently healthy cisgender women between the ages of 18 and 30 years who self-identified their race as Black or African American. Screening procedures ensured participants were free of evidence of clinical sleep disorders or diagnosed conditions known to affect sleep (e.g. depression, insomnia, sleep apnea), did not take any medications or supplements known to affect sleep or vitamin D metabolism, did not work night-shift work, did not have a screening seated resting BP >140 / >90 mmHg, were not taking hypertensive medications or other medications known to affect BP, were non-obese (body mass index ≤30 kg/m^2^), and non-smoking. Additionally, participants were excluded if they had a history of any major chronic diseases or conditions, including cardiometabolic, cardiorespiratory, renal, autoimmune, gastrointestinal, or cancerous conditions, as well as a recent history of coronavirus disease 2019 infection (< 60 days) or vaccination (< 14 days). Participants were also excluded if they reported hyper-/hypocalcemia, hyper-/hypothyroidism, hyper-/hypoparathyroidism, or a history of kidney stones.

### Study protocol

Screening procedures required participants to report to the laboratory during the morning hours (between 07:00 and 11:00 a.m.) following an overnight fast, without caffeine for ≥12 hours, without alcohol, exercise, or vitamins/supplements for ≥24, and without over-the-counter medications or anti-inflammatory drugs for ≥72 hours prior to the visit. The date of assessment, by season, was recorded to account for seasonal variation in sun exposure. After written informed consent was obtained, participants underwent fasted intravenous blood sampling for the assessment of serum [25(OH)D], a complete blood count, basic metabolic panel, and a lipid panel (LabCorp Testing Services). During the screening visit, participants were also asked to report on personal and parental medical history, gynecological history, current medication use including hormonal contraception, vitamin or supplement use, weekly caffeine intake, subjective sleep quality (Pittsburgh Sleep Quality Index; PSQI [24]), sleep hygiene (Sleep Hygiene Index [SHI] [25]), estimated hours of moderate-vigorous physical activity (MVPA) per week (International Physical Activity Questionnaire-Short Form), self-report skin phenotype (Fitzpatrick Skin Phenotype Scale [26, 27]), as well as report on educational attainment and seven social determinants of health (SDoH) on 5-point Likert scales transformed into a composite score ranging from 7–35, which included access to quality education, housing, healthcare, and nutrition, financial security, community acceptance, and stressful experiences over the past 12 months. Participants also underwent screening for insomnia (Insomnia Severity Index score ≥ 15) [28] and sleep apnea (STOP-bang score ≥ 3) [29]. Seated resting BP was assessed in triplicate for screening purposes only (Omron 5 Series, BP7200), height and weight were measured for the calculation of BMI, and body fat percentage was estimated via bioelectrical impedance analysis (Tanita TBF-300A, Arlington Heights, IL).

Eligible participants were then equipped with an accelerometer that they were scheduled to wear on their non-dominant wrist for 14 consecutive days and nights for the estimation of habitual sleep metrics (Philips Actiwatch Spectrum Plus; Philips-Respironics, Inc.). On the final night of sleep monitoring and immediately prior to returning to the lab (day 15), participants completed an overnight urine collection (first morning void). Participants were scheduled to return to the laboratory on day 15.

Participants who successfully completed baseline sleep monitoring and who had serum [25(OH)D] < 30 ng/ml at screening were eligible to enroll in the 8-week vitamin D supplementation intervention. Thus, the vitamin D supplementation intervention includes a subset of participants who completed cross-sectional assessments, which was required for participation in the intervention (*described in detail below under “Intervention Protocol”*). Menstrual cycle was not controlled for throughout the study due to feasibility limitations.

### Device-estimated sleep health metrics

Sleep health metrics were estimated via wrist accelerometry using the Actiwatch Spectrum Plus as previously described [30]. In short, participants were instructed to wear the Actiwatch continuously on the non-dominant wrist for 14 days and nights, in conjunction with completing a standardized daily sleep diary [31]. To estimate habitual sleep health metrics, a conservative minimum of 10 days and nights of wear were required for inclusion in analyses [32]. Data were collected in 30-second epochs and processed by a single trained investigator (M.N.D) using both participants’ standardized sleep diaries and Philips Actiware software (version 6.1.0). Nights with >1 hour of missing data were considered invalid [33]. Rest intervals were identified using a standardized protocol that incorporates sleep diaries, activity levels, and “lights out” [33, 34]. Sleep–wake scoring was based on the medium threshold setting for sleep/wake detection using the algorithm provided by the manufacturer. For each night of wear, sleep duration was estimated as the total time scored as sleep between sleep onset and sleep offset. Sleep efficiency was calculated as total sleep time divided by total time in bed dedicated to sleep, expressed as a percentage. Mean values were generated to characterize habitual sleep duration and sleep efficiency for each participant over the 14-day wear period. Sleep duration regularity was calculated as the SDSD. Sleep timing was operationalized as the timing of sleep onset (clock time at start of each nocturnal sleep period) and sleep timing regularity was calculated as the SOSD. Five BLW were excluded from cross-sectional sleep-onset timing regularity analyses due to the fact that their sleep monitoring spanned over daylight savings time change (Mar. 12, 2023, N = 2; Nov. 5, 2023, N = 3) and four BLW were excluded from intervention sleep-onset timing regularity analyses due to daylight savings time change (Mar. 12, 2023, N = 1; Nov. 5, 2023, N = 2; Mar. 10, 2024, N = 1). For all other sleep metrics, sleep the night of daylight savings time change was excluded from analyses for those participants.

### Nighttime light exposure estimates

Light at night (LAN) was derived from the Actiwatch as mean white light intensity (lux) during each sleep period and nightly values were averaged over the monitoring period [35]. The Philips Actiwatch is able to accurately monitor the temporal patterning of light, although absolute lux values derived from the Actiwatch have been shown to underestimate calibrated photometer-derived lux values in a highly linear fashion [36]. The highly linear association between Actiwatch-derived lux values and photometer-derived lux values allow for accurate within- and between-subject comparisons in light exposure [36].

### Daytime activity estimates

To provide an estimate of daily physical activity, average daily total activity counts (TAC) were derived from the Actiware Software. Average daily TAC is a global measure of physical activity captured by accelerometers and a proxy for the total volume of physical activity performed [37]. Rest / sleep intervals were excluded from the calculation of daily TAC and days with <10 hours of wear time were excluded from analyses [38].

### Overnight urine collection for quantification of melatonin

Participants were provided with a urine collection container and on the morning immediately after the final night of sleep monitoring (day 15) participants were instructed to complete an at-home urine collection for the assessment of overnight 6-sulfatoxymelatonin (aMT6s) excretion, normalized to urine creatinine concentration. Participants were instructed to discard their last urinary void of the evening, right before bedtime, and then collect all voids that occur between sleep onset and sleep offset, as well as their first morning void after awakening, prior to returning to the lab that same morning. Urine was gently mixed by inversion, and 1 ml aliquots were frozen at -80°C for later analysis of aMT6s excretion (TECAN IBL International GmbH, Melatonin-Sulfate Urine ELISA, REF# RE54031, Hamburg, Germany), normalized to urinary creatinine concentration (StressMarq Biosciences, Inc., Urine Creatinine Detection Kit, Catalog# SKT-200, Victoria, BC Canada). Both aMT6s and creatinine were quantified according to manufacturer specifications. All samples were assayed in duplicate. Melatonin-sulfate (aMT6s) intra-assay coefficient of variation (CV) was <12.2% and inter-assay CV was <14.9%. Urinary melatonin: creatinine ratio (ng/mg) was used for all analyses to represent nighttime urinary aMT6s excretion normalized to creatinine.

Importantly, epidemiological studies on both men and women show a significant relationship between nocturnal plasma melatonin concentration, peak nocturnal melatonin concentration, and first morning void urinary aMT6s concentration. Additionally, the sensitivity and specificity of ELISA to quantify melatonin has been shown to be comparable to radioimmunoassay, the most widely used method of determining melatonin concentrations in human urine [39, 40].

### Intervention protocol

**Figure 1:**
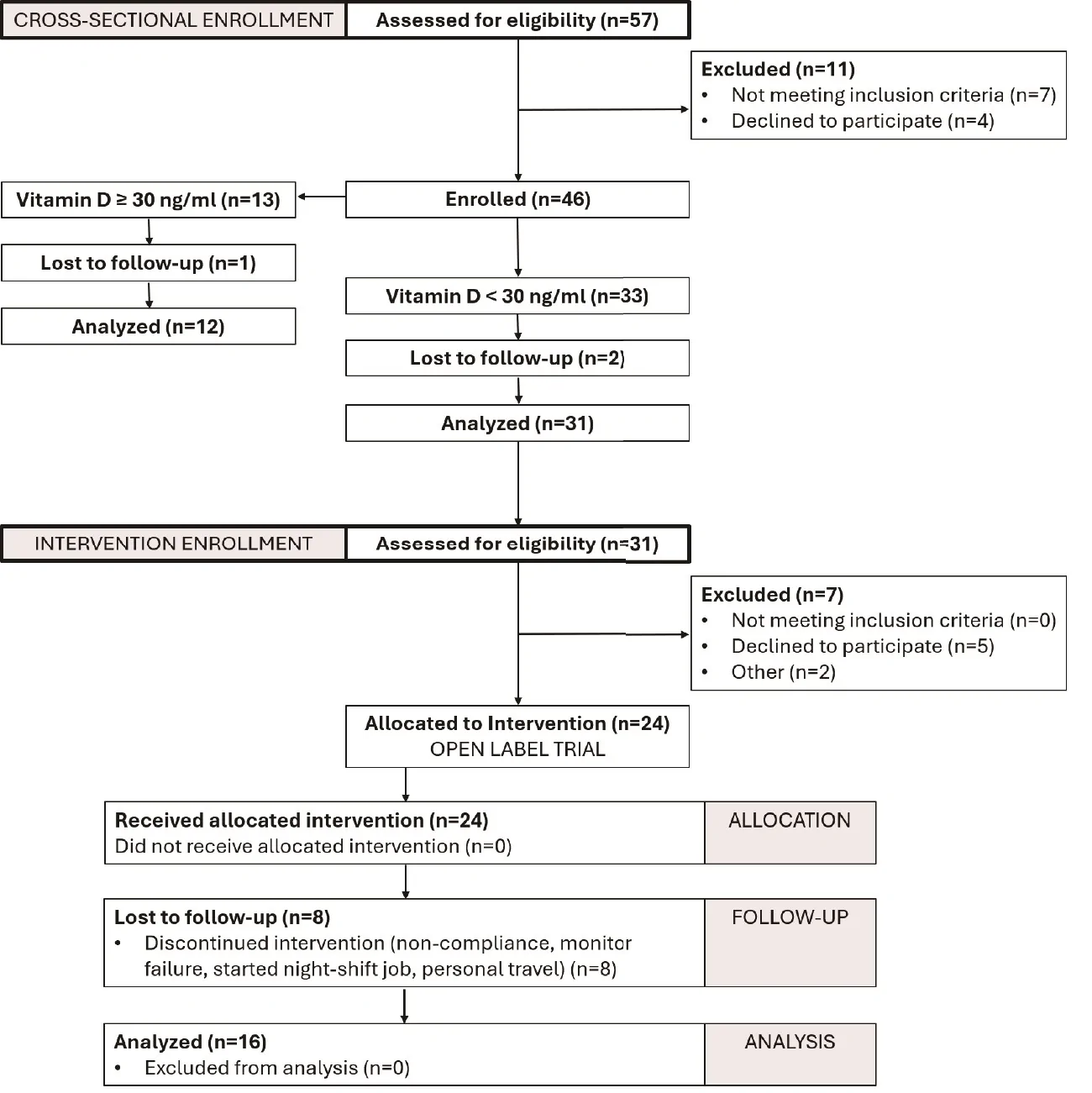
Consolidated standards of reporting trials (CONSORT) flow diagram.

Eligible participants were vitamin D insufficient and deficient, defined as serum [25(OH)D] < 30 ng/ml, and previously completed cross-sectional analyses within the 2 weeks prior to initiating the 8-week vitamin D supplementation intervention (see Fig. 1). Participants were incentivized to enroll in the intervention with free vitamin D supplements, regular monitoring of circulating vitamin D concentrations, and compensation of $120 upon completion. The vitamin D intervention was designed as a single-arm (no placebo group) open-label clinical trial; thus no blinding occurred, and all participants were assigned to the same vitamin D treatment. Prior to initiating the intervention, participants were asked to provide a spot urine sample to confirm pregnancy status via an over the counter hCG detection test (AccuMed Biotech, Houston, TX) administered by a member of the research staff. Participants were initially provided 4-weeks of pills in a 30-day pill organizer to consume prior to returning to the laboratory at week 4 for a mid-intervention venous blood draw to ensure compliance in taking the vitamin D supplements. At week 4 participants were provided with a pre-programmed Actiwatch, a urine collection container, and the final 4-weeks of pills to consume prior to returning to the laboratory at week 8. Each pill was compounded by a local pharmacy to contain 5000 international units (IU) of cholecalciferol (vitamin D3) in white powder form, encapsulated in gelatin derived from porcine and bovine products (Saveway Compounding Pharmacy in Newark, DE). Daily supplementation with 5000 IU of vitamin D3 was chosen based on prior evidence demonstrating that this protocol was able to increase serum [25(OH)D] from an average of ~14 ng/ml at baseline to ~36 ng/ml at week 4 and to ~44 ng/ml at week 8 in adults 18-68-years-old [41]. From these data, we expected that participants with starting serum [25(OH)D] < 30 ng/ml who consumed the supplements as directed would reach final serum vitamin D concentrations between 40 and 60 ng/ml, all reaching a vitamin D sufficiency (≥ 30 ng/ml) within 8 weeks, independent of starting serum [25(OH)D].

Throughout the intervention participants were instructed to consume one vitamin D3 pill per day with food during the morning hours. If a pill was missed, participants were instructed to consume two pills the next day. Participants were asked to leave any unconsumed pills in the container so that compliance could be accurately calculated. A member of the research staff contacted participants weekly throughout the intervention to encourage compliance. Compliance rate was calculated at week 8 as (number of pills consumed / number of pills provided) * 100. Upon return to the laboratory at week 4 and week 8 participants underwent fasted intravenous blood sampling during the morning hours (between 07:00 and 11:30 a.m.) for the assessment of serum [25(OH)D] and blood calcium concentrations (LabCorp Testing Services). Participants were also instructed to wear the Actiwatch for the 14 consecutive days and nights (weeks 7 and 8 of overall intervention) (*as described above*) and complete an overnight urine collection on day 14-15 of the Actiwatch wear-period (day 56 of overall intervention) (*as described above*) immediately prior to returning to the laboratory at week 8. In the laboratory at week 8 participants were also re-administered the PSQI to assess any changes in self-report sleep duration and sleep quality throughout the intervention.

### Covariates

Season of testing and information on SDoH were obtained at screening. Average daily Actiwatch-derived TAC was obtained from 14 days of sleep monitoring. These covariates were selected on a theoretical basis as each of these variables have an established association with either vitamin D [42] or sleep health [43–47] and thus may influence the interrelatedness of these variables cross-sectionally.

### Statistical analyses

**Figure 2:**
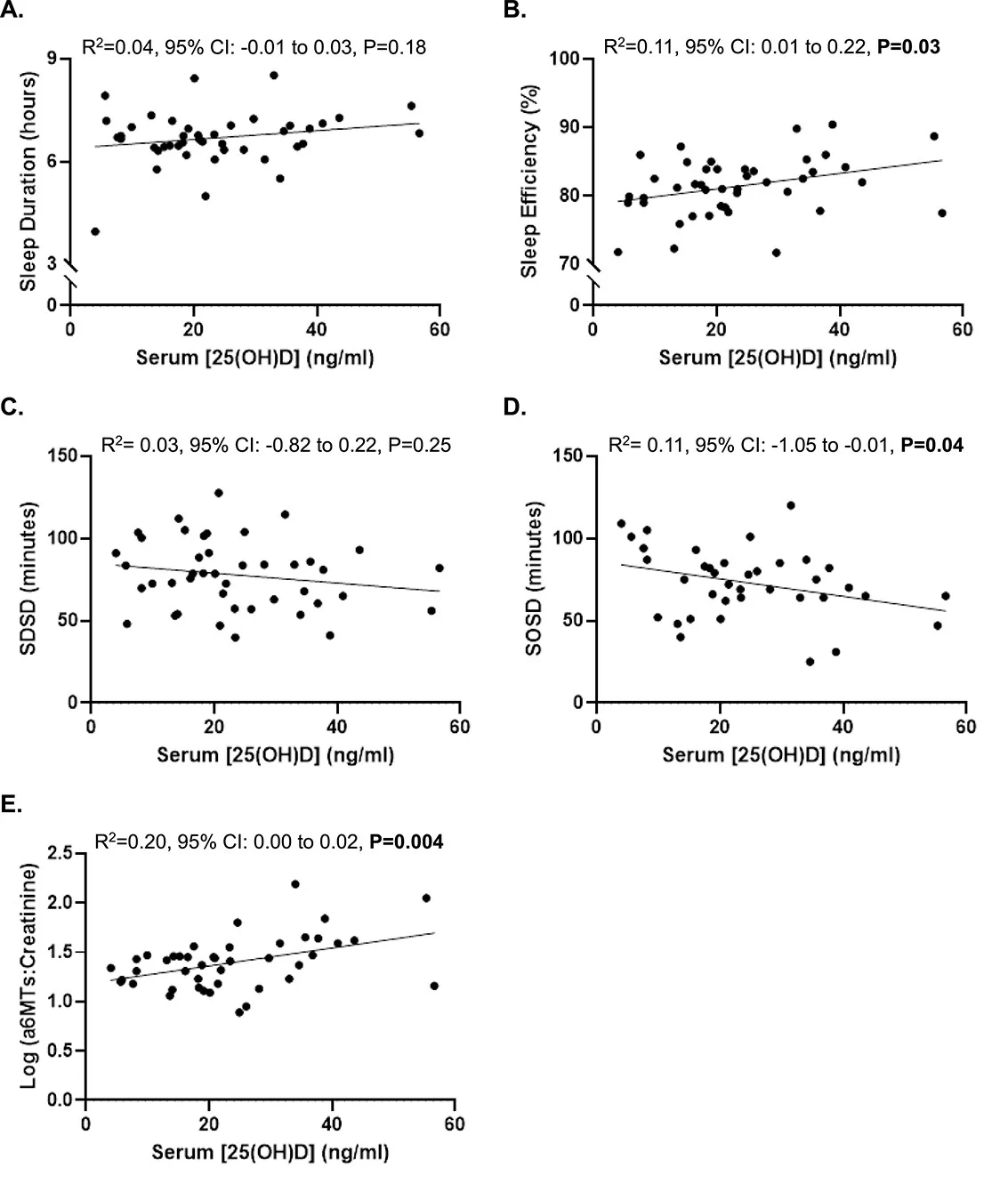
Cross-sectional associations between serum [25(OH)D] and device-estimated sleep duration (A), sleep efficiency (B), SDSD (C), and SOSD^‡^ (D), and between serum [25(OH)D] and log(aMT6s:Creatinine) (E) in young adult black women (N = 43). ^‡^SOSD was assessed in a subset of black women (N = 38). Associations were statistically evaluated via linear regression and *p*-values in **bold** indicate significance set a priori at α ≤ 0.05. aMT6s, urinary 6-sulfatoxymelatonin; R^2^, coefficient of determination; CI, confidence interval; SDSD, sleep duration standard deviation; SOSD, sleep onset standard deviation.

Normality was confirmed for serum [25(OH)D] and sleep metrics using Shapiro–Wilk tests of normality allowing for parametric statistical testing. Summary statistics were used to characterize our sample and separate linear regression models were performed to test cross-sectional associations between serum [25(OH)D] and sleep metrics. aMT6s:creatine concentrations were non-normally distributed; thus raw aMT6s:creatine data were log-transformed prior to testing associations with serum [25(OH)D] and device-estimated sleep metrics (Fig. 2). For participants that completed the intervention, paired sample *t*-tests, Cohen’s d effect sizes, and 95% confidence intervals (CI) were used to compare values from baseline to week 8. Pearson’s *r* correlations were also performed to compare the absolute change (Δ) in serum [25(OH)D] and the Δ in sleep metrics from baseline to week 8.

Testing for potential covariates that may influence the association between serum [25(OH)D] and sleep metrics were performed using separate linear regression models of each sleep metric and multicollinearity was not detected between [25(OH)D] and selected covariates. Linear regression results are presented as R [2], unstandardized *B* values, and 95% confidence intervals (CIs).

A priori power analysis revealed that a total sample size of N = 43 would provide us with at least 80% power to detect a large effect (d = 1.2) at baseline and a total sample size of N = 18 would provide us with at least 80% power to detect a large effect (d = 1.2) across the intervention. Based on preliminary enrollment, this calculation was performed under the assumption that 42% of individuals that enrolled would be vitamin D sufficient (N = 18), 58% of individuals that enrolled would be Vitamin D insufficient/deficient (N = 25), and that 72% of Vitamin D the insufficient/deficient individuals would complete the full 8-week intervention (N = 18). In anticipation of participant dropout and/or noncompliance during the monitoring period, we accounted for a 16% dropout rate. Thus, our target enrollment was N = 52 participants to achieve a final cross-sectional sample size of N = 43 and an intervention sample size of N = 18.Analyses were performed with Microsoft Excel, GraphPad Software (Version 10.2, San Diego, CA), and *jamovi* (Version 2.3.26) [Computer Software], Sydney, Australia. Statistical significance was set a priori at α ≤ 0.05. Summary statistics are presented as means ± standard deviation (SD) or *n* (%) for continuous and categorical variables, respectively.

## Results

### Participants

For cross-sectional baseline assessments, 57 participants were screened for eligibility. Seven participants were deemed ineligible for participation (BMI > 30 kg/m^2^, N = 4; age > 30 years, N = 3), four participants decided not to participate during consenting procedures, and three participants discontinued the study prior to completion. Thus, 43 participants completed cross-sectional assessments and were included in the final cross-sectional analyses. Thirty-one participants had serum [25(OH)D] < 30 ng/ml and were therefore eligible for the intervention. The Consolidated Standards of Reporting Trials (CONSORT) flow diagram is shown in Fig. 1.

### Cross-sectional analyses

Participant characteristics are displayed in Table 1. Overall, 12 participants were vitamin D sufficient ([25(OH)D] ≥30 ng/ml), 12 participants were considered vitamin D insufficient ([25(OH)D] between 20.0-29.9 ng/ml), and 19 participants were considered vitamin D deficient ([25(OH)D] < 20.0 ng/ml). Thirty participants (70%) did not meet clinical sleep duration recommendations (≥ 7 hours per night [4]), 35 participants (81%) did not meet sleep efficiency recommendations (≥ 85% [3]), and 15 participants (35%) had PSQI scores >5 indicating poor subjective sleep quality [48]. Additionally, 12 participants (28%) had SDSD >90 minutes, and seven participants (16%) had SOSD >90 minutes, which have been shown to be associated with >2-fold increased risk of incident CVD in a representative sample of U.S. older adults [5]. Nineteen participants were currently enrolled in an undergraduate degree program, seven participants reported having a bachelor’s degree, and five participants reported having earned a master’s degree. Twenty-five women were naturally cycling, and 18 women were on some form of hormonal birth control (oral: 12; hormonal IUD: 3; Depo-Provera: 1; implant: 2). In addition, all participants were premenopausal and not currently pregnant, and all but one participant were nulliparous.

**Table 1:** Cross-sectional participant characteristics

Participant Characteristics	Mean ± SD or *n* (%)	Range
N	43	–
Age, years	22 ± 3	18–30
BMI, kg/m^2^	23.7 ± 3.6	16.6–30.0
Body Fat, %	29.7 ± 7.7	13.2–42.6
Systolic BP, mmHg	115 ± 7	97–131
Diastolic BP, mmHg	73 ± 7	61–87
Season of Testing, *n* (%)		
Winter	15 (48)	–
Spring	4 (13)	–
Summer	3 (10)	–
Fall	9 (29)	–
Skin Phenotype, score	4 ± 1	3–6
Caffeine, drinks/week	3 ± 3	0–14
Self-Report MVPA, hrs/week	6.6 ± 5.5	0.0–23.8
Sleep Hygiene Index, score	18 ± 7	9–36
**Fasted Blood Values**	**Mean ± SD or *n* (%)**	**Range**
25(OH)D, ng/ml	23.4 ± 12.6	4.0–56.6
Calcium, mg/dl	9.4 ± 0.2	8.9–9.8
Glucose, mg/dl	85 ± 5	75–97
eGFR, mL/min/1.73	110 ± 14	82–136
Total Cholesterol, mg/dl	164 ± 28	95–217
HDL Cholesterol, mg/dl	64 ± 14	36–102
LDL Cholesterol, mg/dl	88 ± 26	38–142
Triglycerides, mg/dl	60 ± 23	24–138
RBC Count, x10E6/uL	4.52 ± 0.43	3.76–5.72
Hemoglobin, g/dL	12.6 ± 1.0	10.2–15.3
Hematocrit, %	38.8 ± 3.1	32.3–48.3
WBC Count, x10E3/uL	5.2 ± 1.3	3.4–8.3

Values presented as mean ± standard deviation for continuous variables or *n* (%) for categorical variables. BMI, body mass index; BP, blood pressure; eGFR, estimated glomerular filtration rate; HDL-C, high-density lipoprotein cholesterol; LDL-C, low-density lipoprotein cholesterol; MVPA, moderate-vigorous physical activity; RBC, red blood cell; WBC, white blood cell; 25(OH)D, 25-hydroxyvitamin D.

On average, participants wore the Actiwatch for 13 ± 2 days. Unadjusted associations between serum [25(OH)D] and device-estimated sleep metrics and Log(aMT6s:creatinine) are displayed in Fig. 2. Specifically, linear regressions demonstrate significant cross-sectional associations for [25(OH)D] with sleep efficiency (β = 0.11, *p* = .03; %) and SOSD (β = -0.53, *p* = .04; minutes), but no associations with sleep duration or SDSD (*p* > .18). After multivariable adjustment for the season of testing, physical activity, and SDoH, serum [25(OH)D] remained not a significant predictor of sleep duration (B = 0.01, 95% CI = -0.01 to 0.03, *p* = .47) and SDSD (B = -0.38 hours, 95% CI = -1.02 to 0.26, *p* = .23). However, multivariable adjustment diminished the significant associations between serum [25(OH)D] and sleep efficiency (B = 0.10, 95% CI = -0.03 to 0.23, *p* = .13) and serum [25(OH)D] and SOSD (B = -0.49, 95% CI = -1.09 to 0.10, *p* = .10).

Average global PSQI score was 5 ± 3 (range: 0 – 14). Serum [25(OH)D] was not cross-sectionally associated with global PSQI score (B = 0.01 score, 95% CI = -0.05 to 0.07, *p* = .68), subjective sleep duration (B = 0.02 hours, 95% CI = -0.01 to 0.05, *p* = .12), or subjective sleep efficiency (B = 0.00 %, 95% CI = -0.14 to 0.14, *p* = .99), nor any of the PSQI sub-component scores (*data not shown*). Of note, further analyses revealed a significant association between subjective and Actiwatch-derived sleep duration (Pearson’s r = 0.40, *p* = .01), but no association between subjective and Acti-watch-derived sleep efficiency (Pearson’s r = 0.04, *p* = .80). Interestingly, Log(aMT6s:creatinine) was not associated with any device-estimated sleep metrics including sleep duration (B = -0.21 hours, 95% CI = -1.13 to 0.71, *p* = .65), sleep efficiency (B = 3.64%, 95% CI = -1.30 to 8.59, *p* = .15), SDSD (B = -13.50 minutes, 95% CI = -37.91 to 10.90, *p* = .27) and SOSD (B = -9.42 minutes, 95% CI = -34.88 to 16.04, *p* = .46) and findings were not different if raw aMT6s:creatinine values were used (*data not shown*). Log(aMT6s:creatinine) was also not associated with global PSQI score (B = 0.07 score, 95% CI = -2.82 to 2.97, *p* = .96) or self-report sleep duration (B = -0.76 hours, 95% CI = -2.20 to 0.68, *p* = .30).

### Intervention analyses

**Figure 3:**
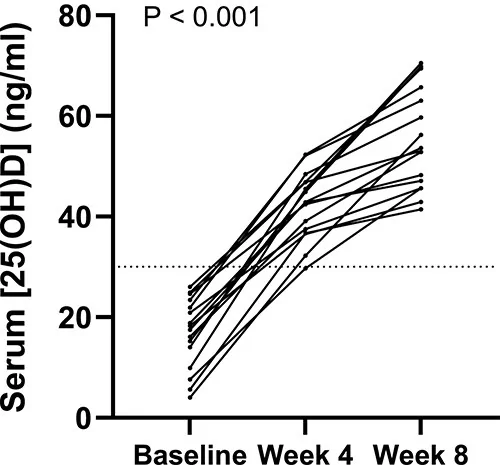
Individual changes in serum [25(OH)D] for participants (N = 16) throughout the 8-week oral vitamin D3 supplementation intervention with 5000 IU of vitamin D3 per day. Changes in serum [25(OH)D] were statistically evaluated from baseline to week 8 via a paired-sample t-test, although data are shown for baseline, week 4 (mid-intervention assessment) and week 8. Significance was set a priori at α ≤ 0.05. Horizontal dashed line represents vitamin D sufficiency cut-off for serum [25(OH)D] at 30 ng/ml. Mean ± standard deviation values for [25(OH)D] across the intervention are as follows: baseline, 16.8 ± 7.0 ng/ml; week 4, 42.3 ± 6.6 ng/ml; week 8 55.3 ± 10.0 ng/ml.

Sixteen participants completed the full 8-week vitamin D supplementation intervention. Baseline characteristics among participants that completed the intervention (N = 16), discontinued the intervention (N = 8), or declined to participate in the intervention (N = 5) are presented in Table S1. Average vitamin D3 supplement compliance rate assessed by pill count was 99 ± 3% (range: 91%–100%). The average total amount of vitamin D3 consumed during the 8-week intervention was 273 750 ± 11 030 IUs (range: 250 000 – 290 000 IU). Individual changes in serum [25(OH)D] throughout the intervention are displayed in Fig. 3. Importantly, at week 8 of the intervention all participants achieved sufficient serum [25(OH)D] (>30 ng/ml). However, we did not observe any effect of vitamin D supplementation on device-estimated sleep metrics, Log(aMT6s:creatinine), or self-report sleep (Table 2). Further, no associations were observed between the change in serum [25(OH)D] and the change in device-estimated sleep duration (r = 0.08, *p* = .77), sleep efficiency (r = 0.03, *p* = .90), SDSD (r = -0.02, *p* = .93), SOSD (r = -0.09, *p* = .79), or Log(aMT6s:creatinine) (r = 0.08, *p* = .78) from baseline to week 8.

**Table 2:** The effect of an 8-week daily oral vitamin D supplementation intervention on device-estimated, physiological, and self-report sleep metrics among participants with baseline serum [25(OH)D] < 30 ng/ml (N = 16)

Actiwatch Values	Baseline	Week 8	Cohen’s d	95% CI	*p*-Value
Sleep Duration, hrs	6.3 ± 0.9	6.3 ± 0.9	−0.05	−0.54 to 0.44	0.89
Sleep Efficiency, %	80.6 ± 3.9	80.8 ± 4.0	0.05	−0.44 to 0.54	0.85
SDSD, min	80.2 ± 21.6	81.3 ± 20.3	0.05	−0.44 to 0.54	0.83
SOSD, min^‡^	78.4 ± 17.7	84.3 ± 20.3	0.38	−0.21 to 0.96	0.21
Average LAN, lux	0.98 ± 1.69	0.55 ± 0.60	−0.24	−0.72 to 0.26	0.35
Average daily TAC, counts	214 160 ± 39 143	204 570 ± 41 611	−0.27	−0.78 to 0.25	0.32
**Urinary Melatonin**	**Baseline**	**Week 8**	**Cohen’s d**	**95% CI**	***p*-Value**
aMT6s:Creatinine^‡^	23.5 ± 14.0	23.7 ± 12.2	0.03	−0.50 to 0.55	0.90
Log(aMT6s:Creatinine)^‡^	1.31 ± 0.24	1.31 ± 0.25	0.03	−0.49 to 0.56	0.93
**Self-Report Sleep (PSQI)**	**Baseline**	**Week 8**	**Cohen’s d**	**95% CI**	***p*-Value**
Sleep Duration, hrs	6.5 ± 0.9	6.6 ± 1.1	0.08	−0.41 to 0.57	0.75
Sleep Efficiency, %	92.4 ± 7.9	93.8 ± 7.2	0.12	−0.41 to 0.64	0.66
Global PSQI score	5.9 ± 3.0	5.4 ± 2.8	−0.15	−0.64 to 0.35	0.56
Comp_1 score (Quality)	1.1 ± 0.7	1.1 ± 0.5	−0.15	−0.67 to 0.38	0.58
Comp_2 score (Latency)	1.3 ± 1.1	1.1 ± 1.0	−0.15	−0.67 to 0.38	0.58
Comp_3 score (Duration)	1.1 ± 0.6	0.9 ± 0.6	−0.19	−0.71 to 0.35	0.50
Comp_4 score (Efficiency)	0.1 ± 0.5	0.1 ± 0.5	0.00	−0.52 to 0.52	1.00
Comp_5 score (Disturbance)	1.1 ± 0.4	1.0 ± 0.4	−0.27	−0.80 to 0.27	0.34
Comp_6 score (Medications)	0.1 ± 0.4	0.1 ± 0.5	0.00	−0.52 to 0.52	1.00
Comp_7 score (Daytime Dysfunction)	1.0 ± 1.0	1.0 ± 0.9	0.00	−0.52 to 0.52	1.00

^‡^, SOSD and aMT6s:Creatinine were evaluated in a subset of participants (SOSD, N = 12; aMT6s:Creatinine, N = 14). Values presented as means ± standard deviation. Paired sample *t*-tests were used to assess changes in sleep metrics from baseline to week 8. Significance was set a priori at α ≤ 0.05. aMT6s, urinary 6-sulfatoxymelatonin; LAN, light at night; PSQI, Pittsburgh Sleep Quality Index; SDSD, sleep duration standard deviation; SOSD, sleep onset standard deviation; TAC, total activity counts.

## Discussion

Cross-sectionally, a majority of the BLW in this study were characterized as having vitamin D insufficiency/deficiency as well as short sleep duration and low sleep efficiency as assessed by wrist actigraphy. Main cross-sectional findings from this study are that serum [25(OH)D] was positively associated with device-estimated sleep efficiency, inversely associated with SOSD, and positively associated with urinary aMT6s excretion, although serum [25(OH)D] was not associated with device-estimated sleep duration or SDSD. Interestingly, among vitamin D insufficient/deficient BLW that completed the 8-week vitamin D supplementation intervention, increasing serum [25(OH)D] did not have an effect on any device-estimated sleep metrics or urinary aMT6s excretion at the end of the intervention.

Population-based data indicate that BLW have the highest rates of vitamin D deficiency and experience shorter and more disturbed sleep compared with women of other races [15, 49]. In our sample of 43 young BLW that completed cross-sectional baseline assessments, 72% had suboptimal serum vitamin D concentrations. Sleep health was also compromised in this sample as 70% of BLW achieved less than the recommended ≥7 hours of sleep per night, 81% achieved less than the recommended ≥85% sleep efficiency, 28% had a SDSD >90 minutes, and 16% had a SOSD >90 minutes as determined by 14-days of wrist actigraphy. These estimates of poor sleep are higher than those observed in our laboratory’s previous study of college-enrolled young adults, where 51% obtained <7 hours of sleep per night and 27% had a SDSD >90 minutes [50]. Further, in the present study 35% of BLW had global PSQI scores >5 which is indicative of poor sleep quality [48].

Large cross-sectional studies support associations between serum [25(OH)D] concentration and sleep health. Data from the Multi-Ethnic Study of Atherosclerosis demonstrated that vitamin D deficiency was significantly associated with shorter device-estimated sleep duration in fully adjusted models, with this association being strongest among Black adults compared with other racial/ethnic groups [18]. Our laboratory recently extended these findings to younger populations, reporting that each 1 ng/ml increase in serum [25(OH)D] was significantly associated with a 0.02 hour increase in sleep duration, a 0.17% increase in sleep efficiency, a 0.50 minute decrease in SDSD, and a 0.95 minute decrease in SOSD as estimated via 14 days of wrist actigraphy in healthy young and early mid-life adults [20].

Cross-sectional findings from the present study indicate that serum [25(OH)D] was positively associated with device-estimated sleep efficiency and SOSD, although no associations were observed with sleep duration or SDSD. These associations were attenuated after multivariable adjustment for season of testing, Actiwatch-derived physical activity, and SDoH suggesting that these factors may confound the association between serum [25(OH)D] and sleep in this sample and may offer insight for future investigations examining these associations. Regardless, our data demonstrate a high prevalence of vitamin D insufficiency/deficiency as well as a high prevalence of short sleep duration and low sleep efficiency estimated via wrist actigraphy among participants. Thus, due to the homogeneity of our study population and our limited sample size, it becomes challenging to decipher the impact of serum [25(OH)D] on sleep outcomes cross-sectionally. Accordingly, our 8-week vitamin D supplementation intervention among vitamin D insufficient/deficient BLW offers further insight regarding the potential effect of vitamin D on device-estimated sleep health outcomes.

To the best of our knowledge, ours is the first investigation of the effect of vitamin D supplementation on device-estimated sleep outcomes (duration, efficiency, regularity) among vitamin D insufficient/deficient young adult BLW. Previous studies have only evaluated this in the context of self-reported sleep metrics. However, both device-estimated and self-report (PSQI) findings from the present study do not align with the limited previous double-blind randomized placebo-controlled trials indicating improvements in *subjective* sleep health following oral vitamin D supplementation in young adults. For example, in a study comprised of young and middle-aged adults with sleep disorders, bi-weekly supplementation with 50 000 IU of vitamin D for 8 weeks significantly improved (decreased) global PSQI score and increased self-report sleep duration as compared with a placebo group [51]. In addition, among vitamin D deficient patients with insomnia, daily supplementation with 1500 IU of vitamin D for 10 weeks significantly improved insomnia symptoms as assessed by the Insomnia Severity Index as compared with a placebo group, despite only a small average increase in serum [25(OH)D] from 15 ng/ml to 19 ng/ml [52]. Several other studies have been conducted in participants experiencing chronic pain, which have all also demonstrated a significant improvement in subjective sleep outcomes following vitamin D supplementation, although likely attributed to a reduction in pain symptoms in response to the vitamin D treatment [53–56]. Thus, the present study is the first to explore the effect of vitamin D supplementation using both device and self-reported estimates of sleep in vitamin D insufficient/deficient young adult BLW who were free of evidence of clinical sleep disorders. In contrast to our hypothesis, we did not demonstrate any influence of an 8-week oral vitamin D supplementation intervention on sleep outcomes in the present study despite a significant and robust increase in serum [25(OH)D]. However, investigating this in young BLW was especially relevant as they represent a population at elevated risk for vitamin D deficiency, short sleep duration, and low sleep efficiency.

Interestingly, in the present study we observed a significant positive cross-sectional association between serum [25(OH)D] and urinary aMT6s excretion, but no change in urinary aMT6s excretion following the vitamin D supplementation intervention. On a cellular level, vitamin D has been implicated in the homeostatic regulation of sleep as it has been shown to increase activity of the enzyme tryptophan hydroxylase 2 (TPH2), which aids in the downstream production of melatonin [16]. TPH2 contains two vitamin D response element sequences that are associated with transcriptional activation of the gene [57] and treatment of human brain glioblastoma/ astrocytoma (U87 MG) cells with 1,25(OH)D (the active metabolite of vitamin D) was found to significantly increase TPH2 mRNA in a dose-dependent manner [16]. In addition, treatment of adipose-derived stem cells with 1,25(OH)D was found to elicit synchronization of *BMAL1* and *Per2* gene expression, demonstrating a profile similar to adipose-derived stem cells synchronized by a serum shock, implicating vitamin D in the circadian process of sleep regulation [17]. In our sample we observed significant cross-sectional associations between serum [25(OH)D] and sleep efficiency and sleep onset timing (SOSD), but failed to detect any significant associations between urinary aMT6s excretion and device-estimated sleep outcomes. Although cross-sectional findings support a potential link between serum [25(OH)D] and urinary aMT6s excretion, no change in urinary aMT6s excretion was observed following 8 weeks of vitamin D supplementation. We speculate that acutely increasing serum [25(OH)D] may not elicit observable effects on melatonin production that are detectable in vivo.

### Experimental considerations

This pilot investigation is the first to evaluate the effect of vitamin D on device-estimated sleep metrics and urinary aMT6s excretion using combined cross-sectional and longitudinal study designs in young BLW. This study design uniquely allowed us to directly compare cross-sectional findings with the effect of an 8-week vitamin D supplementation intervention. Importantly, all participants that completed the 8-week vitamin D supplementation were vitamin D insufficient/deficient at baseline and were highly compliant with the intervention, allowing all participants to reach sufficient vitamin D status by week 8. Our study design also allowed us to confirm supplement compliance and observe an initial, robust increase in serum [25(OH)D] at week 4, prior to re-initiating sleep monitoring. In addition, we employed a rigorous 14 days of wrist actigraphy for the evaluation of habitual sleep metrics using a highly reproducible sleep watch that has been validated against polysomnography in healthy adults [34]. Still it should be noted that wrist actigraphy is limited by its specificity for detecting quite wakefulness and may over-estimate sleep efficiency and total sleep time [58], thus other methods of objective sleep detection may be needed to validate our findings. Lastly, despite our null findings regarding the effect of vitamin D insufficiency/deficiency on device-estimated sleep metrics, this work remains impactful as cardiovascular-related research conducted in BLW is especially limited [59–61].

This study also has some limitations. Sleep is a modifiable health behavior, and a majority of our participants were enrolled in college, thus ongoing social and lifestyle factors may have contributed to unchanged habitual sleep metrics across the intervention, hindering any observable effects of vitamin D on sleep health outcomes. Further, especially given our small sample size, the characteristics of the participants in this pilot study may not be representative of other young BLW from different regions in the U.S. or with different education levels, as all of the BLW in this study were either currently enrolled in a bachelor’s degree program or had obtained a bachelor’s degree or higher. Indeed, race is a social construct and the contributors to racial health disparities are likely multifactorial and rooted in systemic and structural racism leading to socioeconomic disadvantages as well as implicit biases in medicine and healthcare [62]. In our study the significant associations observed between [25(OH)D] and sleep efficiency and SOSD were abolished after multivariable adjustment for season of testing, physical activity, and SDoH, which seemed to be mostly driven by season of testing and should be accounted for in future investigations. We also conducted this investigation over a relatively short (8 week) supplementation period in young, otherwise healthy BLW, which may have contributed to our null findings. It is possible that the positive effects of vitamin D sufficiency on sleep health metrics in otherwise healthy adults may require a longer period of correction; thus, future work should consider the impact of long-term or regular vitamin D supplement use on sleep heath metrics in young BLW, as well as across the lifespan. Finally, the route of obtaining vitamin D may be an important consideration for future work, particularly in the context of sleep health. Specifically, higher daytime sun exposure may be a strong mechanistic link driving the cross-sectional association between higher serum vitamin D concentrations and better sleep health, while simultaneously explaining the absence of improvements in sleep health outcomes with oral vitamin D supplementation alone.

## Conclusions

In this pilot investigation we observed a high prevalence of vitamin D insufficiency/deficiency, short sleep duration, and low sleep efficiency among young adult BLW. Cross-sectional findings from this study support a potential link between serum [25(OH)D] and device-estimated sleep efficiency and SOSD, as well as urinary aMT6s excretion in young adult BLW. However, longitudinal findings from this study do not support the ability of an 8-week oral vitamin D3 supplementation intervention to have an effect on device-estimated sleep metrics, urinary aMT6s excretion, or subjective sleep duration/quality in young adult BLW. Future work should consider a longer vitamin D supplementation intervention period as well as other physiological, social, or behavioral influences of sleep disturbances in BLW early in life, as mitigating the prevalence of these CVD risk factors in young adulthood may aid in the primordial prevention of CVD and other adverse health-related outcomes later in life.

## Supplementary Material

Suppl_Data_Final_zpag096

## Data Availability

The data that support the findings of this study are available from the corresponding author upon reasonable request.
