# Supplementary material for: A pilot study investigating the effect of serum 25-hydroxyvitamin D on device-estimated sleep metrics in young black women: a single-arm open-label clinical trial
Source: Sleep Adv. 2026 Sep 1;7(3):zpag096. doi: 10.1093/sleepadvances/zpag096 (PMC13592166; doi:10.1093/sleepadvances/zpag096)
Supplement: Supplementary file 1 [file suppl_data_final_zpag096.docx]

**A Pilot Study Investigating the Effect of Serum 25-Hydroxyvitamin D on Device-Estimated Sleep Metrics in Young Black Women:**

**A Single-Arm Open-Label Clinical Trial**

Michele N. D’Agata^1^, Krista M. Szymanski^1^, Freda Patterson^2^, Mackenzie L. Rattigan^1^, Alexs A. Matias^1^, David G. Edwards^1^, Christopher R. Martens^1^, Sushant M. Ranadive^3^, Melissa A. Witman^1^.

^1^Department of Kinesiology and Applied Physiology

College of Health Sciences

University of Delaware

Newark, DE, USA

^2^Department of Health Behavior and Nutrition Science

College of Health Sciences

University of Delaware

Newark, DE, USA

^3^Department of Kinesiology

School of Public Health

University of Maryland

College Park, MD, USA

**Corresponding Author:**

Melissa A. Witman, PhD

University of Delaware

Department of Kinesiology & Applied Physiology

540 S. College Avenue

Newark, DE, 19716

302-831-6256

mwitman@udel.edu

**Supplemental Data**

| **Table S1.** | **Completed Intervention** | | **Discontinued Intervention** | | **Declined to Participate** | |
| --- | --- | --- | --- | --- | --- | --- |
| **Participant Characteristics** | **Mean ± SD or *n* (%)** | **Range** | **Mean ± SD or *n* (%)** | **Range** | **Mean ± SD or *n* (%)** | **Range** |
| N | 16 | - | 8 | - | 5 | - |
| Age, years | 22 ± 4 | 18 - 30 | 22 ± 2 | 19 - 25 | 22 ± 3 | 19 - 28 |
| BMI, kg/m^2^ | 24.8 ± 4.0 | 17.3 - 30.0 | 24.2 ± 2.8 | 21.2 - 28.6 | 24.6 ± 3.3 | 21.0 - 28.5 |
| Body Fat, % | 30.9 ± 7.9 | 18.4 - 42.5 | 30.3 ± 8.6 | 20.0 - 42.6 | 30.0 ± 8.3 | 16.6 - 39.1 |
| Systolic BP, mmHg | 116 ± 9 | 104 - 131 | 114 ± 4 | 108 - 119 | 115 ± 6 | 105 - 122 |
| Diastolic BP, mmHg | 74 ± 8 | 61 - 87 | 74 ± 5 | 68 - 84 | 73 ± 6 | 66 - 81 |
| Season of Baseline Testing, *n* (%) | | |  |  |  |  |
| Winter | 8 (50) | - | 3 (38) | - | 2 (40) | - |
| Spring | 1 (6) | - | 2 (25) | - | 1 (20) | - |
| Summer | 1 (6) | - | 1 (12) | - | 1 (20) | - |
| Fall | 6 (38) | - | 2 (25) | - | 1 (20) | - |
| Skin Phenotype, score | 5 ± 1 | 3 - 6 | 5 ± 1 | 3 - 6 | 5 ± 1 | 4 - 6 |
| Caffeine, drinks/week | 3 ± 3 | 0 - 10 | 2 ± 2 | 0 - 4 | 1 ± 1 | 0 - 3 |
| Self-Report MVPA, hrs/week | 8.0 ± 7.3 | 0.0 - 23.8 | 6.7 ± 4.7 | 2.3 - 14.7 | 7.3 ± 3.5 | 3.8 - 12.5 |
| Sleep Hygiene Index, score | 18 ± 5 | 10 - 28 | 18 ± 8 | 9 - 29 | 13 ± 4 | 9 - 19 |
| **Fasted Blood Values** | **Mean ± SD or *n* (%)** | **Range** | **Mean ± SD or *n* (%)** | **Range** | **Mean ± SD or *n* (%)** | **Range** |
| 25(OH)D, ng/ml | 16.8 ± 7.0 | 4.0 - 26.0 | 15.9 ± 6.4 | 5.8 - 23.3 | 22.2 ± 6.5 | 14.2 - 29.7 |
| Calcium, mg/dl | 9.4 ± 0.3 | 8.9 - 9.8 | 9.4 ± 0.3 | 9.1 - 9.8 | 9.4 ± 0.3 | 9.0 - 9.7 |
| Glucose, mg/dl | 86 ± 6 | 78 - 97 | 86 ± 2 | 84 - 90 | 82 ± 6 | 75 - 90 |
| eGFR, mL/min/1.73 | 112 ± 13 | 93 - 133 | 106 ± 12 | 91 - 128 | 105 ± 17 | 82 - 123 |
| Total Cholesterol, mg/dl | 163 ± 27 | 114 - 217 | 169 ± 41 | 95 - 216 | 166 ± 25 | 127 - 194 |
| HDL Cholesterol, mg/dl | 68 ± 15 | 36 - 102 | 57 ± 16 | 39 - 89 | 75 ± 8 | 64 - 84 |
| LDL Cholesterol, mg/dl | 83 ± 21 | 51 - 135 | 98 ± 35 | 38 - 142 | 79 ± 27 | 43 - 115 |
| Triglycerides, mg/dl | 59 ± 22 | 30 - 107 | 71 ± 33 | 24 - 138 | 53 ± 17 | 38 - 80 |
| RBC Count, x10E6/uL | 4.43 ± 0.35 | 3.76 - 5.09 | 4.53 ± 0.33 | 4.16 - 5.00 | 4.64 ± 0.30 | 4.11 - 4.86 |
| Hemoglobin, g/dL | 12.4 ± 1.0 | 10.2 - 13.7 | 12.7 ± 1.1 | 11.8 - 14.2 | 12.9 ± 0.7 | 11.9 - 13.6 |
| Hematocrit, % | 38.1 ± 2.5 | 32.3 - 41.5 | 38.0 ± 2.4 | 35.8 - 41.9 | 40.4 ± 1.8 | 38.4 - 42.5 |
| WBC Count, x10E3/uL | 5.0 ± 1.3 | 3.6 - 8.3 | 5.8 ± 1.3 | 4.4 - 7.6 | 5.4 ± 0.9 | 4.3 - 6.7 |

**Table S1 (supplemental).** Baseline characteristics among participants that completed the intervention (N=16), discontinued the intervention (N=8), or declined to participate in the intervention (N=5). Values presented as mean ± standard deviation for continuous variables or *n* (%) for categorical variables. BMI, body mass index; BP, blood pressure; eGFR, estimated glomerular filtration rate; HDL-C, high-density lipoprotein cholesterol; LDL-C, low-density lipoprotein cholesterol; MVPA, moderate-vigorous physical activity; RBC, red blood cell; WBC, white blood cell; 25(OH)D, 25-hydroxyvitamin D.
